# Principal Curves for Statistical Divergences and an Application to Finance

**DOI:** 10.3390/e20050333

**Published:** 2018-05-02

**Authors:** Ana Flávia P. Rodrigues, Charles Casimiro Cavalcante

**Affiliations:** Department of Teleinformatics Engineering, Federal University of Ceará, Fortaleza-CE 60440-900, Brazil

**Keywords:** principal curves, information geometry, deformed exponential, finance application

## Abstract

This paper proposes a method for the beta pricing model under the consideration of non-Gaussian returns by means of a generalization of the mean-variance model and the use of principal curves to define a divergence model for the optimization of the pricing model. We rely on the *q*-exponential model so consider the properties of the divergences which are used to describe the statistical model and fully characterize the behavior of the assets. We derive the minimum divergence portfolio, which generalizes the Markowitz’s (mean-divergence) approach and relying on the information geometrical aspects of the distributions the Capital Asset Pricing Model (CAPM) is then derived under the geometrical characterization of the distributions which model the data, all by the consideration of principal curves approach. We discuss the possibility of integration of our model into an adaptive procedure that can be used for the search of optimum points on finance applications.

## 1. Introduction

In their seminal paper [1], Hastie and Stuetzle proposed a notion of principal curves as an elegant and geometric non-linear generalization of factor models as the principal component analysis. A principal curve has the property of self-consistence in the sense that it passes through the middle of the data set representing a sample of some random variable. More precisely, any point of the curve coincides with the expected value of the data projected on it. This is a direct consequence of the fact that a principal curve f is critical for the variance of the Euclidean distance between the data and any locally defined perturbation of f. In particular, a straight line is a principal curve if and only if its direction is an eigenvector of the covariance matrix of *z*, where *z* stands for the a vector containing the observed data.

The original idea by Hastie and Stuetzle has been developed into relevant improvements, applications and extensions. We point out however that the criticality of a principal curve is usually defined in terms of the Euclidean distance. Hence, although f itself could represent non-Euclidean features of the model, some underlying least-squares approach is still in force. Our main contribution here is to rephrase the notion of principal curves (and, more generally, of principal *p*-submanifolds) in terms of a general statistical divergence which replaces the Euclidean divergence, that is, the variance used in the original definition.

Considering statistical divergences as Kullback–Leibler or Bregman divergence allows us to deal with random variables with probabilities given by exponential and deformed exponential distributions. In the context of exponential and ϕ-exponential statistical families, straight lines are replaced by affine geodesics and the Hessian of the cumulant function plays the role of a generalized covariance.

As highlighted by Naudts, deformed exponentials play a central role in the foundations of the Generalized Thermostatistics formulated by Tsallis [2,3] and collaborators. This new approach to Thermodynamics has been evolved along the last two decades in a wide range of applications to complex systems, particularly in Finance [4,5,6,7]. Indeed, Naudts’ work established deep and fruitful connections between Statistical Physics and Information Geometry [8,9,10,11]. For instance, both Rényi’s and Tsallis’ entropies are described by Naudts in terms of statistical divergences in the family of *q*-exponential distributions that includes *q*-Gaussian distributions, defined in details by Plastino and Vignat [11,12,13,14,15]. The analytic and geometric features of deformed exponentials suggest that they are well suited to model non-normally distributed returns of contingent claims. In this direction, for instance, a non-Gaussian option pricing theory has been successfully proposed in terms of diffusion processes associated to *q*-Gaussian distributions [4,7,16,17,18]. Other related developments are summarized in [6,19].

In [20,21], the authors elaborated some preliminary results towards a theory of portfolio optimization in the context of deformed exponentials. One of the cornerstones of the modern Finance Theory, the classical Markowitz’s mean-variance model of portfolio selection, relies on the assumptions that the returns of assets are normally distributed and that the investor preferences are described by constant risk-aversion utility function. Some works have dealt with other variations of the portfolio optimization problem. For instance, Zagrodny [22] proposed a convex programming solution to portfolio selection by considering the Hilbert space and a reinsurance approach. In the multi-objective model, Sawik [23] used the expected return as the performance metric and expected worst-case return to measure the risk and providing good interpretation about the consideration of the variance of the risks.

The traditional criticism to the normality assumption in Markowitz’s theory raises the need of alternative models for dealing with non-Gaussian distributions. This question has been addressed since then under different methods. In [24,25], Nock et al. extended the Markowitz’s model to the wider family of exponential distributions, replacing the mean-variance by a mean-divergence model. Bregman divergences replace the variance as risk measures for non-Gaussian distributions, eventually encompassing information from higher order momenta. On the other hand, since statistical divergences define geometric notions on the statistical manifold of exponential distributions, their method has a geometric interpretation in terms of a steepest descent by the natural gradient of the risk premium [26,27,28].

In [20], the authors proposed a model of portfolio selection of financial assets that explores the non-additivity and non-normality aspects of Tsallis’ Thermostatistics. More precisely, they have extended the mean-divergence model in [24,25] to deformed exponentials families.

In the sequel, the authors formulated [21] a generalization of beta pricing models adapted to a mean-divergence portfolio selection [29,30,31]. In particular, it is presented an extension of Capital Asset Pricing Model (CAPM) flexible enough to be applied for financial returns with deformed exponential distributions. The method relies on a geometric approach to the classical mean-variance analysis developed by LeRoy and Werner [32] and Luenberger [33] (see also [34]). The main results in [20,21] are summarized in Section 3.

This paper is structured as follows. In Section 2, we define the generalized notion of principal curve and principal submanifold in the geometric context of a given statistical divergence. The earlier contributions for portfolio selection and asset pricing in the case of financial returns distributed according deformed exponential probability densities are schematically resumed in Section 3. In Section 4, we apply the generalized notion of principal submanifolds and the correspondent version of the principal component analysis to obtain an explicit expression of optimal principal portfolios is provided in Section 5.

## 2. Statistical Divergences and Principal Curves

Let M be a space of random variables z=z(s) whose probability distributions are given by densities lying in a *n*-dimensional statistical manifold

$$S=\{p\left(s,\vartheta \right):\vartheta \in U\subset R^{n}\},$$

where $\vartheta =(\vartheta_{1},\ldots ,\vartheta_{n})$ are statistical parameters ranging in some open subset *U* of the *n*-dimensional Euclidean space $R^{n}$. Let *D* be a given statistical divergence in S. Given a curve f:Λ⊂R→M, a projection of *z* on the trace of f is a point $f(\lambda_{*})$, for some $\lambda_{*}\in \Lambda$, such that

(1) $$D\left(z|f\left(\lambda_{*}\right)\right)=\underset{\lambda \in \Lambda }{\inf}D\left(z|f\left(\lambda \right)\right).$$

In the following, we suppose that such a projection exists and it is unique for any curve f:Λ→M we are going to consider. Under this assumption, we denote

(2) $$\pi_{f}\left(z\right)=f\left(\lambda_{*}\right).$$

In this notation, we propose the following variational notion of principal curve relative to *D*:

**Definition** **1.** *A curve f:Λ→M is a* principal curve *in (M,D) if*

(3) $$D\left(z|\pi_{f}\left(z\right)\right)=\underset{s\in (-\epsilon ,\epsilon )}{\inf}D\left(z|\pi_{f_{s}}\left(z\right)\right)$$

*for all one-parameter family of curves $f_{s}:\Lambda \to M$, s∈(−ϵ,ϵ), such that $f_{0}=f$.*

Recall that a statistical divergence *D* determines a dually flat structure in M for which affine geodesics are parameterized as straight lines of the form

f(λ)=a+λu,λ∈Λ,

where a and u are constant vectors. By definition, the projection of the random variable *z* on a principal curve f minimizes the divergence among the projections on curves close to f. Projections satisfy a Pythagorean theorem, one of the fundamental results in Information Geometry that can be stated as follows.

**Theorem** **1** (Theorem 1.2 and Theorem 1.3, [26])**.** *Given o,z,w∈M such that that the dual affine geodesic connecting z and w is orthogonal to the affine geodesic connecting w and o, the following generalized Pythagorean relation holds*

(4) D(z|o)=D(w|o)+D(z|w).

*Similarly, if the affine geodesic connecting z and w is orthogonal to the dual affine geodesic connecting w and o, we have the dual relation*

(5) $$D^{*}\left(z|o\right)=D^{*}\left(w|o\right)+D^{*}\left(z|w\right),$$

*where $D^{*}$ is the dual divergence. The dual divergence is defined with respect to the dual connection as defined in [26].*

In view of this proposition, it is natural to draw our attention to one-parameter families of affine geodesics in M.

**Theorem** **2.** *An affine geodesic in (M,D) is a principal curve with respect to one-parameter families of affine geodesics if and only if its direction is an eigenvector of the Fisher metric $G=\nabla^{2}D$ associated to D.*

**Proof.**  Denote by ∇D and $\nabla^{2}D$, respectively, the differential and Hessian of *D* with respect to the second variable. Hence, we have for a fixed s∈(−ϵ,ϵ) that

$$\begin{array}{c} 0=\frac{d}{d\lambda }D\left(z|f\left(s,\lambda \right)\right)=\frac{d}{d\lambda }D\left(z|f\left(s,\lambda \right)\right)=\langle \nabla D\left(z|f\left(s,\lambda \right)\right),\frac{\partial f}{\partial \lambda }\rangle \end{array}$$

where the derivative is computed at the critical value $\lambda =\lambda_{*}\left(s\right)$ and for a fixed value of *s*, $f_{s}$ is a geodesic parameterized by λ which we can write $f_{s}\left(\lambda \right)=f\left(s,\lambda \right)$.

Denote

$$c\left(s\right)=f(s,\lambda_{*}\left(s\right)),\;s\in \left(-\epsilon ,\epsilon \right).$$

Note that

$$c^{\prime }\left(s\right)=\frac{\partial f}{\partial s}\left(s,\lambda_{*}\left(s\right)\right)+\frac{\partial f}{\partial \lambda }\left(s,\lambda_{*}\left(s\right)\right)\frac{d\lambda_{*}}{ds}.$$

Hence, we obtain

$$\begin{array}{c} \frac{d}{ds}\langle \nabla D\left(z|c\left(s\right)\right),\frac{\partial f}{\partial \lambda }\left(s,\lambda_{*}\left(s\right)\right)\rangle =\langle \nabla D\left(z|c\left(s\right)\right),\frac{d}{ds}\frac{\partial f}{\partial \lambda }\left(s,\lambda_{*}\left(s\right)\right)\rangle \\ \;\;\;\;+c^{\prime }{\left(s\right)}^{⊤}\nabla^{2}D\left(z|c\left(s\right)\right)\frac{\partial f}{\partial \lambda }\left(s,\lambda_{*}\left(s\right)\right)=\langle \nabla D\left(z|c\left(s\right)\right),\frac{d}{ds}\frac{\partial f}{\partial \lambda }\left(s,\lambda_{*}\left(s\right)\right)\rangle \\ \;\;\;\;+{\left(\frac{\partial f}{\partial s}\left(s,\lambda_{*}\left(s\right)\right)+\frac{\partial f}{\partial \lambda }\left(s,\lambda_{*}\left(s\right)\right)\frac{d\lambda_{*}}{ds}\right)}^{⊤}\nabla^{2}D\left(z|c\left(s\right)\right)\frac{\partial f}{\partial \lambda }\left(s,\lambda_{*}\left(s\right)\right). \end{array}$$

We may write

$$f\left(s,\lambda \right)=f\left(\lambda \right)+sv\left(\lambda \right)+O\left(s^{2}\right),$$

where

$$v\left(\lambda \right)=\frac{\partial f}{\partial s}\left(0,\lambda \right)$$

is the variational field that corresponds to f. Thus, we have

$$\frac{\partial f}{\partial \lambda }\left(s,\lambda_{*}\left(s\right)\right)=f^{\prime }\left(\lambda_{*}\left(s\right)\right)+sv^{\prime }\left(\lambda_{*}\left(s\right)\right)+O\left(s^{2}\right)$$

and

$$\frac{d}{ds}{|}_{s=0}\frac{\partial f}{\partial \lambda }\left(s,\lambda_{*}\left(s\right)\right)=f^{\prime \prime }\left(\lambda_{*}\right)\frac{d\lambda_{*}}{ds}{|}_{s=0}+v^{\prime }\left(\lambda_{*}\left(s\right)\right).$$

If f=f(0,·) is a critical curve, we have

$$\begin{array}{cc} 0 & =\frac{d}{ds}{|}_{s=0}\langle \nabla D\left(z|c\left(s\right)\right),\frac{\partial f}{\partial \lambda }\left(s,\lambda_{*}\left(s\right)\right)\rangle =\langle \nabla D\left(z|f\left(\lambda_{*}\right)\right),\frac{d}{ds}\frac{\partial f}{\partial \lambda }\left(0,\lambda_{*}\right)\rangle \\ & +{\left(v\left(\lambda_{*}\right)+\frac{d\lambda_{*}}{ds}{|}_{s=0}f^{\prime }\left(\lambda_{*}\right)\right)}^{⊤}\nabla^{2}D\left(z|f\left(\lambda_{*}\right)\right)f^{\prime }\left(\lambda_{*}\right). \end{array}$$

We conclude that

$$\begin{array}{c} {\left(v\left(\lambda_{*}\right)+\frac{d\lambda_{*}}{ds}{|}_{s=0}f^{\prime }\left(\lambda_{*}\right)\right)}^{⊤}\nabla^{2}D\left(z|f\left(\lambda_{*}\right)\right)f^{\prime }\left(\lambda_{*}\right)\\ \;\;\;\;+\langle \nabla D\left(z|f\left(\lambda_{*}\right)\right),f^{\prime \prime }\left(\lambda_{*}\right)\frac{d\lambda_{*}}{ds}{|}_{s=0}+v^{\prime }\left(\lambda_{*}\left(s\right)\right)\rangle =0. \end{array}$$

Setting $f^{\prime \prime }\left(\lambda \right)=0$ and $v^{\prime }\left(\lambda \right)=0$, one gets

$$\begin{array}{c} {\left(v\left(\lambda_{*}\right)+\frac{d\lambda_{*}}{ds}{|}_{s=0}f^{\prime }\left(\lambda_{*}\right)\right)}^{⊤}\nabla^{2}D\left(z|f\left(\lambda_{*}\right)\right)f^{\prime }\left(\lambda_{*}\right)=0. \end{array}$$

Since $v(\lambda_{*})$ can be arbitrarily chosen in such a way that $v(\lambda_{*})$ and $f^{\prime }\left(\lambda_{*}\right)$ are linearly independent, we conclude there exists μ∈R such that

(6) $$\nabla^{2}D\left(z|f\left(\lambda_{*}\right)\right)f^{\prime }\left(\lambda_{*}\right)=\mu f^{\prime }\left(\lambda_{*}\right).$$

This means that $f^{\prime }\left(\lambda_{*}\right)$ is an an eigenvector of the Fisher information metric at the point $f(\lambda_{*})$

(7) $$G_{f(\lambda_{*})}=\nabla^{2}D\left(z|f\left(\lambda_{*}\right)\right)$$

associated to the divergence *D*. This finishes the proof. ☐

A result concerning principal submanifolds similar to Theorem 2 follows easily as a scholia of its proof: we may consider the projection of the random variable *z* onto a *p*-dimensional affine submanifold in M parameterized by a smooth map $f:\Lambda \subset R^{p}\to M$ whose differential has rank *p*. The submanifold f(Λ) is principal with respect to families of affine submanifolds if and only if it is spanned by *p* geodesics whose velocities are linearly independent and are eigenvectors of the Hessian matrix $G=\nabla^{2}D$ at the projection point.

A fundamental example of divergence is the Euclidean $L^{2}$-norm

$$D_{\mathrm{euc}}\left(z|w\right)=\frac{1}{2}{\left|z-w\right|}^{2},\;z,w\in M$$

on which is based both the least-squares method and the principal component analysis. In their seminal work [1], Hastie and Stuetzle proved that a Euclidean straight line is a principal curve with respect to their definition if and only if its direction is an eigenvector of the covariance matrix of the random variable *z*.

Now, we obtain an extension of this result by Hastie and Stuetzle valid in the context of non-Euclidean statistical divergences. In our setting, the role of the covariance matrix is played by its non-Euclidean and non-Gaussian counterpart, namely the Hessian matrix $\nabla^{2}K$, where *K* is the cumulant generating function.

**Corollary** **1.** *Let K be a convex function in S and D be the Bregman divergence in M determined by K. Then an affine geodesic is a principal curve with respect to one-parameter families of affine geodesics in M if and only if its direction is an eigenvector of the Hessian of K.*

**Proof.**  This follows directly from Theorem 2 once we have observed that the Fisher metric in this case coincides with the Hessian of *K*. This is however a well-known fact that may be deduced easily from the definition of the Bregman divergence itself as

(8) D(z|w)=K(z)−K(w)−〈∇K(w),z−w〉.

For details, we refer the reader to [26]. ☐

In the sequel, we are going to consider more general examples, not necessarily quadratic. For instance, we may fix the Kullback–Leibler divergence

$$D_{KL}\left(z\left(\cdot ,\vartheta \right)|w\left(\cdot ,\vartheta^{\prime }\right)\right)=\int p\left(s,\vartheta \right)\log\left(\frac{p(s,\vartheta )}{p(s,\vartheta^{\prime })}\right)\;ds$$

or, more generally, the relative ϕ-entropy associated to ϕ-exponential distributions.

## 3. The Space of Financial Assets

From now on, M stands for the linear span of financial assets traded in a securities market. More precisely, every point in M corresponds to the payoff *z* of a contingent claim at a fixed time, say t=1, a random variable

z=z(s),

where *s* are the states of the world with probability distribution specified by some density p(s;ϑ). Recall that ϑ is the distribution parameter of a family of probability distributions in a *n*-dimensional statistical manifold S.

In the following, we will consider the statistical manifold of ϕ-exponential probability densities

$$p\left(s,\vartheta \right)=\exp_{\phi }\left(\langle T\left(s\right),\vartheta \rangle -K\left(s,\vartheta \right)\right)\;p_{0}\left(\vartheta \right),\;\vartheta \in R^{n},$$

where *T* is a sufficient statistics of the random variable z(s) and *K* is the moment generating function. Here, $p_{0}$ is a fixed reference density and $\exp_{\phi }$ is the ϕ-exponential defined as the inverse function of the ϕ-logarithm [8,9]

$$\log_{\phi }\left(t\right)=\int_{1}^{t}\frac{1}{\phi (s)}\;ds,$$

where ϕ:(0,+∞)→(0,+∞) is a strictly positive, nondecreasing and continuous real function. A particular case of this deformed exponential is given by the *q*-exponential function

$$\exp_{q}\left(t\right)={\left(1+\left(1-q\right)t\right)}^{\frac{1}{1-q}}$$

with q>0, which corresponds to set $\phi \left(t\right)=t^{q}$, Hence, the *q*-logarithm is defined by

$$\log_{q}\left(t\right)=\int_{1}^{t}\frac{1}{s}ds=\frac{1}{1-q}\left(t^{1-q}-1\right).$$

The moment generating function *K* defines a Bregman divergence given by

D(z|w)=K(z)−K(w)−〈∇K(z),z−w〉.

where the probability distributions of z(s) and w(s) are, respectively, given by the densities p(s,ϑ) and $p(s,\vartheta^{\prime })$.

### 3.1. Deformed Exponentials and Portfolio Selection

Setting ϕ(t)=t, one gets the family of exponential distributions, in particular multivariate Gaussian distributions. For this family, Nock et al. [24,25] represented the key concepts of Portfolio Selection theory in terms of the moment generating function and the associated Bregman divergence. More precisely, they proved that, for constant absolute risk aversion (CARA) utility functions, the certainty equivalent and risk premium of risky assets are, respectively, given by

$$C=\frac{1}{a}\left(K\left(z\left(\cdot ,\vartheta \right)\right)-K\left(w\left(\cdot ,\vartheta^{\prime }\right)\right)\right)$$

and

$$\Pi =\frac{1}{a}D\left[z\left(\cdot ,\vartheta \right)\right)\left|w\left(\cdot ,\vartheta^{\prime }\right)\right],$$

where a>0 is a risk-aversion parameter. Hence, they extended the classical mean-variance portfolio selection to a general mean-divergence model for which an optimal allocation α is a solution of the minimization problem

$$\min_{\alpha }\left(\langle \nabla K\left(\vartheta -a\alpha \right),\alpha \rangle +\frac{1}{a}D_{\phi }\left(\vartheta \right|\left(\vartheta -a\alpha \right)\right).$$

In the particular case of Gaussian distributed returns, they easily recover the classical Markowitz’s optimal portfolio allocation vector

$$\alpha =\frac{\Sigma^{-1}1}{1^{⊤}\Sigma^{-1}1},$$

where Σ is the variance-covariance matrix of the returns on the assets.

In [20], the authors extended this approach to ϕ-exponential distributions, in particular to *q*-exponential distributions. They proved that the optimal portfolio for their extended mean-divergence model is given in terms of the cumulant function by

(9) $$\alpha =\frac{\nabla^{2}K{\left(\vartheta \right)}^{-1}1}{1^{⊤}\nabla^{2}K{\left(\vartheta \right)}^{-1}1}\cdot$$

Note that the Hessian of the (convex) function *K* is positive-definite and plays the role of the variance-covariance matrix in the Gaussian case. In the particular case of *q*-Gaussian distributions [14], the optimal allocation portfolio is given by

(10) $$\alpha =\frac{\Sigma_{q}^{-1}1}{1^{⊤}\Sigma_{q}^{-1}1}$$

where

(11) $$\Sigma_{q}=\gamma_{q}C_{q,n}^{1-q}{\left|\Sigma \right|}^{\frac{1-q}{2}}\Sigma$$

with

(12) $$\gamma_{q}=\frac{1}{2}\left((n+4)-(n+2)q\right)$$

and

(13) $$C_{q,n}=\left\{\begin{array}{c} \frac{\Gamma \left(\frac{1}{q-1}-\frac{n}{2}\right)\sqrt{\pi }}{\Gamma (\frac{1}{q-1})}{\left(\frac{1}{q-1}\right)}^{\frac{n}{2}}{\left((n+4)-(n+2)q)\right)}^{\frac{n}{2}},\;\mathrm{for}\;1<q<\frac{n+4}{n+2},\\ \frac{\Gamma \left(\frac{2-q}{1-q}\right)\sqrt{\pi }}{\Gamma (\frac{2-q}{q-1}+\frac{n}{2})}{\left(\frac{1}{1-q}\right)}^{\frac{n}{2}}{\left((n+4)-(n+2)q)\right)}^{\frac{n}{2}},\;\mathrm{for}\;q<1. \end{array}\right.$$

Here, |Σ| is the determinant of Σ. We refer the reader to [14] for further details in *q*-multivariate Gaussian distributions. It is evident that one re-obtains the Markowitz’s portfolio for q=1 in Equation (10).

In view of Equation (9), the authors have elaborated in [20] a steepest descent algorithm by the natural (Riemannian) gradient of the risk premium. Some empirical support to the proposed method is provided by comparing the cumulated returns and the evolution of the divergence for optimal portfolios according to the mean-divergence model and the classical one by Markowitz. The numerical evaluations in [20] show the proposal is able to yield better tracking of deep changes in the stock market, such as the ones present in crisis scenarios, and yet produce a higher return than the classical mean-variance strategy.

### 3.2. Mean-Divergence Efficient Frontier

In Markowitz’s model, the optimal portfolio allocation lies in the mean-variance efficient frontier that bounds the feasible set of allowed returns and risks of traded risky portfolios. In [32,33], LeRoy, Werner and Luenberger have developed a geometric approach to the mean-variance analysis in terms of the geometry of orthogonal projections onto a mean-variance efficient frontier. From this approach, they easily deduce an elegant geometric interpretation of the celebrated Capital Asset Pricing Method (CAPM) as well as other factor pricing models.

In [21], the authors have extended the geometric pricing method to general divergence geometries in M instead of the Hilbert space $L^{2}$-norm.

Since *K* is a strictly convex function, its Hessian is positive-definite and then defines a Riemannian metric in M, that is, for each z∈M, we define an inner product in the tangent space $T_{z}M$ by

(14) $$G_{z}=\nabla^{2}K\left(z\right).$$

This metric can be expanded in local coordinates around a fixed reference point o∈M as

(15) $$G_{z}∼\nabla^{2}K\left(o\right)+{o(|z|}^{2}),$$

where quadratic terms are determined in terms of the Riemann curvature of the Riemannian manifold (M,g), see [35].

Denote by $k_{e}$ the expectation kernel, that is, an asset in M that yields the expected payoffs of the assets in M. More precisely,

$$G_{z}\left(k_{e},z\right)=E\left[z\right]$$

for any z∈M. We define the pricing kernel $k_{q}$ as an asset in M that gives the price of any contingent claim z∈M as the expected discounted payoff

$$g\left(k_{q},z\right)=E\left[mz\right]=Q\left(z\right),$$

where *m* is a stochastic discount factor. Here, Q:M→R is the price functional, that is, the present value of the expected returns of the asset, discounted at rate *m*. The existence of this functional is one of the consequences of the Fundamental Theorem of Finance Theory whose key assumption is that there are no arbitrage portfolios in M. For a comprehensive treatment of those fundamentals on Finance, we refer the reader to [32,36].

Denote by E the subspace in M spanned by $k_{e}$ and $k_{q}$. The projection $z^{E}$ of z∈M onto E is defined by

$$D\left(z^{E}|z\right)=\min_{w\in E}D\left(w|z\right).$$

It follows from the generalized Pythagorean Theorem for divergences (Theorem 1) that, fixing a reference point o∈M, one has

(16) $$D\left(z|o\right)=D\left(z^{E}|o\right)+D\left(z|z^{E}\right),\;z\in M^{\prime }.$$

If the case of the divergence given by the Euclidean $L^{2}$-norm in M

$$D_{\mathrm{euc}}\left(z|w\right)=\frac{1}{2}{\left|z-w\right|}^{2}$$

Equation (16) reduces to the Euclidean decomposition

(17) $$E\left[{\left|z\right|}^{2}\right]=E{\left[z\right]}^{2}+\mathrm{var}\left[z\right],$$

where

$$\mathrm{var}\left[z\right]=E\left[{\left(z-E\left[z\right]\right)}^{2}\right]$$

is the variance, the classical risk measure in Portfolio Theory [36,37].

Motivated by the analogy between Equations (16) and (17), the authors proposed in [21] the projection

$$\Pi \left(z\right)=D\left(z|z^{E}\right)$$

as a novel risk measure for assets z∈M. Since it depends on the whole information about the probability densities p(s,ϑ), this measure encodes higher moments of *z* instead of only the variance. Moreover, one easily verifies that Π is the variance in the case of normally distributed returns and Euclidean divergence. Hence, we have defined a risk measure that embodies non-normality and non-Euclidean features of the returns of financial assets and the estimation of their statistical parameters, respectively.

The main result in [21] is that the two reference assets $k_{e}$ and $k_{q}$ determine the efficient frontier for portfolios of assets in M with respect to the risk measure Π. Indeed, we have the following theorem.

**Theorem** **3** (Theorem 2 in [21])**.** *Let $E=\mathrm{span}\{k_{e},k_{q}\}$ the subspace in M spanned by the expectation and pricing kernels. Given z∈M, we have*

$$E\left[z\right]=E\left[z^{E}\right]$$

*and*

$$\Pi \left(z^{E}\right)\leq \Pi \left(z\right)$$

*where $z^{E}$ is the projection of z onto E.*

Since the efficient frontier is spanned by two assets, this last result can be regarded as a non-Gaussian and non-Euclidean version of the two-fund spanning theorem in Finance. Generalizing the mean-variance case, we can prove in the case of ϕ-exponentials that the efficient mean-divergence frontier for portfolio selection is spanned by two portfolios

$$\alpha_{1}=\frac{{\left(\nabla^{2}K\right)}^{-1}1}{1^{⊤}{\left(\nabla^{2}K\right)}^{-1}1}\;\mathrm{and}\;\alpha_{\mu }=\frac{{\left(\nabla^{2}K\right)}^{-1}\mu }{1^{⊤}{\left(\nabla^{2}K\right)}^{-1}\mu },$$

where μ is the desired expected return of the portfolio.

## 4. Generalized Beta Pricing Models and CAPM

Denote by $R_{e}$ and $R_{q}$ the returns of $k_{e}$ and $k_{q}$, respectively. In [21], the authors have proved that the minimum divergence portfolio in M is given by

$$z=R_{e}+\left(1-\beta \right)\left(R_{q}-R_{e}\right)$$

where

$$\beta =-\frac{g(R_{q}-R_{e},R_{e})}{g(R_{q}-R_{e},R_{q}-R_{e})}.$$

A similar expression holds replacing the basic assets $k_{e}$ and $k_{q}$ by two efficient assets $k_{\lambda }$ and $k_{\nu }$ in E such that

(18) $$G(r_{\lambda },r_{\nu })=0.$$

These zero-covariance pair of assets is given by

$$r_{\lambda }=R_{e}+\lambda \left(R_{q}-R_{e}\right)$$

and

$$r_{\nu }=R_{e}+\nu \left(R_{q}-R_{e}\right)$$

where ν is given by

(19) $$\nu =-\frac{G\left(R_{e},R_{e}\right)+\lambda G\left(R_{q}-R_{e},R_{e}\right)}{G\left(R_{q}-R_{e},R_{e}\right)+\lambda G\left(R_{q}-R_{e},R_{q}-R_{e}\right)}.$$

Note that ν is well-defined if and only if $k_{\lambda }$ is not the minimum divergence portfolio in E.

We have obtained in [21] a generalized beta pricing equation involving $k_{\lambda }$ and $k_{\nu }$

(20) $$E\left[z\right]=E\left[r_{\nu }\right]+\beta \left(E\left[r_{\lambda }\right]-E\left[r_{\nu }\right]\right)$$

for assets in z∈M, where the generalized beta coefficient is given by

(21) $$\beta =\frac{G(r,r_{\lambda })}{G(r_{\lambda },r_{\lambda })}\cdot$$

If there exists a risk-free asset 1 with return $R_{f}$ in M, we fix $r_{\nu }=1$ reducing Equation (20) to

(22) $$E\left[z\right]=R_{f}+\beta \left(E\left[r_{\lambda }\right]-R_{f}\right).$$

As in the classical CAPM, we can take $r_{\lambda }$ as the market return $r_{m}$ since it is possible to prove under some assumptions that $r_{m}$ is in the mean-divergence efficient frontier. More precisely, this is the case when every agent in the market has consumption preferences given by a time-separable utility function of the form

(23) $$u\left(c_{0},c_{1}\right)=u_{0}\left(c_{0}\right)+u_{1}\left(E\left[c_{1}\right],G{|}_{c_{1}}\left(c_{1},c_{1}\right)\right)$$

where $u_{1}$ is strictly decreasing with respect to the second variable. Here, $c_{0}$ is the agent’s consumption plan at time t=0 and $c_{1}=c_{1}\left(s\right)$ is a random variable in M that describes the consumption plan of the agent at time t=1.

Under this assumption, we obtained in [21] a generalized CAPM equation

(24) $$E\left[r\right]-R_{f}=\tilde{\beta }\left(E\left[r_{m}\right]-R_{f}\right),$$

where $r_{m}$ is the return of the market portfolio and

(25) $$\tilde{\beta }=\frac{G(r,r_{m})}{G(r_{m},r_{m})}$$

is the generalized beta market. This coefficient measures the generalized covariance between the risk of the asset or portfolio and the market risk. Note that both Equations (20) and (24) define a generalized security market line [32,38].

The Fisher information metric G plays the role here of the covariance matrix. In the particular case when the returns of traded assets are distributed according to a *q*-Gaussian distribution, it holds that

$$G_{z}=\nabla^{2}K\left(z\right)=\Sigma_{q}$$

for every z∈M, where the *q*-variance matrix $\Sigma_{q}$ is defined in Section 3.1.

## 5. Generalized Principal Components Analysis (PCA) and Applications to Finance

The results we have quoted in Section 3.2 and Section 4 indicate that the Hessian information matrix

(26) $$G=\nabla^{2}D$$

plays a central role in the extension of portfolio selection and asset pricing models in the case of non-Gaussian returns. Even under the assumption of normality of the asset returns, G can provide a more accurate risk measure since it is sensitive to higher moments of the underlying probability distributions.

A portfolio composed by *N* risky assets $z_{1},\ldots ,z_{N}$ in M is determined by an allocation vector $\alpha =\left(\alpha_{1},\ldots ,\alpha_{N}\right)\in R^{N}$

(27) αD,

where *D* is the vector of payoffs ${\left(z_{1},\ldots ,z_{N}\right)}^{⊤}$. We assume that the payoffs have probability distributions given by densities $p(s,\vartheta_{i})\in S$, i=1,…,N. The expected return of this portfolio is

$$\mu =\alpha E\left[D\right]=\sum_{i=1}^{N}\alpha_{i}E\left[z_{i}\right]$$

whereas its generalized covariance is given by

$$\pi =G\left(\alpha D,\alpha D\right)=\sum_{i,j=1}^{N}\alpha_{i}G\left(z_{i},z_{j}\right)\alpha_{j}.$$

The matrix

(28) $$G_{ij}:=G\left(z_{i},z_{j}\right)$$

is referred to as the generalized covariance matrix of the assets $z_{1},\ldots ,z_{n}$. Thus, we consider the optimization problem

(29) $$\min_{\alpha }\pi$$

subject to the constraint

(30) $$\alpha \alpha^{⊤}=1.$$

Setting the Lagrangian

$$L=\pi -\lambda_{1}\left(\alpha \alpha^{⊤}-1\right),$$

one easily verifies that the first order necessary condition for the optimal portfolio $F^{\left(1\right)}$ is

(31) $$\sum_{j=1}^{N}G_{ij}F_{j}^{\left(1\right)}=\lambda_{1}F_{i}^{\left(1\right)},$$

that is, $F^{\left(1\right)}$ is an eigenvector of the generalized covariance matrix G relative to the eigenvalue $\lambda_{1}$. Supposing the G has *N* distinct eigenvalues and iterating this same optimization procedure in subspaces orthogonal to the span of the already given eigenvectors, one obtains the principal directions $F^{\left(1\right)},\ldots ,F^{\left(N\right)}$ correspondent to the eigenvalues

$$\lambda_{1}\geq \ldots \geq \lambda_{N}\geq 0.$$

We then define a matrix *R* by

$$z_{j}=\sum_{i=1}^{N}R_{ij}F^{\left(i\right)}$$

in such a way that an arbitrary portfolio’s payoffs may be rewritten as

$$\alpha D=\sum_{j=1}^{N}\left(\sum_{i=1}^{N}\alpha_{j}R_{ij}\right)F^{\left(i\right)}=:\sum_{i=1}^{N}\beta_{i}F^{\left(i\right)}.$$

Next, we restrict ourselves to the projections of portfolios onto the (totally geodesic) affine subspace spanned by the first p<N principal directions $F^{\left(1\right)},\ldots ,F^{\left(p\right)}$, taken as the most significant ones because they represent the largest *p* diagonal elements in the generalized covariance matrix in diagonal form, that is,

$$G_{\mathrm{diag}}=R^{-1}GR.$$

Hence, we obtain a multi-factor linear model of the form

(32) $$\alpha D=\sum_{i=1}^{p}\beta_{i}F^{\left(i\right)}+\varepsilon ,$$

where

$$\varepsilon =\sum_{i=p+1}^{N}\beta_{i}F^{\left(i\right)}$$

satisfies

$$G\left(\varepsilon ,\sum_{i=1}^{p}\beta_{i}F^{\left(i\right)}\right)=0.$$

The expected return of the *p*-principal portfolio

$$\sum_{i=1}^{p}\beta_{i}F^{\left(i\right)}$$

is

$$\sum_{i=1}^{p}\beta_{i}E\left[F^{\left(1\right)}\right]$$

and its generalized variance is given by

$$\sum_{i=1}^{p}\lambda_{i}\beta_{i}^{2}.$$

We claim that the *p*-principal portfolio with expected return $\mu_{*}$ and minimum generalized variance is determined by the weights

(33) $$\beta_{i}={\left(\sum_{j=1}^{p}\frac{E[F^{\left(j\right)}]}{{\sqrt{\lambda }}_{j}}\right)}^{-1}\frac{E[F^{\left(i\right)}]}{\lambda_{i}}.$$

To prove this claim, we denote

$${\tilde{\beta }}_{i}=\sqrt{\lambda_{i}}\beta_{i},\;R_{i}=\frac{1}{{\sqrt{\lambda }}_{i}}E\left[F^{\left(i\right)}\right]$$

and then we set the Lagrangian

$$\frac{1}{2}\sum_{i=1}^{p}{\tilde{\beta }}_{i}^{2}-\nu \left(\sum_{i=1}^{p}{\tilde{\beta }}_{i}R_{i}-\mu_{*}\right)$$

with a constraint given by

$$\sum_{i=1}^{p}\frac{1}{{\sqrt{\lambda }}_{i}}{\tilde{\beta }}_{i}=1.$$

The first order condition is

$${\tilde{\beta }}_{i}=\nu R_{i},$$

for all i=1,…,p. Taking traces and using the constraint condition, one gets

$$\nu \sum_{i=1}^{p}\frac{R_{i}}{{\sqrt{\lambda }}_{i}}=1.$$

We conclude that

(34) $$\beta_{i}={\left(\sum_{j=1}^{p}\frac{R_{j}}{{\sqrt{\lambda }}_{j}}\right)}^{-1}\frac{R_{i}}{\sqrt{\lambda_{i}}}$$

as claimed. In sum, we have proven the following theorem.

**Theorem** **4.** *The p-principal portfolio with minimum generalized variance is given by*

(35) $$\beta_{i}={\left(\sum_{j=1}^{p}\frac{E[F^{\left(j\right)}]}{{\sqrt{\lambda }}_{j}}\right)}^{-1}\frac{E[F^{\left(i\right)}]}{\lambda_{i}},$$

*where $E[F^{\left(i\right)}]$ and $\lambda_{i}$, i=1,…,p, are, respectively, the expected return and the generalized variance of the first p eigenvectors $F^{\left(1\right)},\ldots ,F^{\left(p\right)}$ of the generalized covariance matrix $G=\nabla^{2}D$. This portfolio coincides with the projection of the random variable z=αD over the principal p-dimensional submanifold spanned by the eigenvectors.*
